# Adaptive Finite Element Modeling of Linear Elastic Fatigue Crack Growth

**DOI:** 10.3390/ma15217632

**Published:** 2022-10-30

**Authors:** Abdulnaser M. Alshoaibi, Abdullateef H. Bashiri

**Affiliations:** Mechanical Engineering Department, Jazan University, P.O. Box 114, Jazan 45142, Saudi Arabia

**Keywords:** adaptive mesh, finite element method, linear elastic fracture mechanics, node splitting, crack growth, stress intensity factors

## Abstract

This paper proposed an efficient two-dimensional fatigue crack growth simulation program for linear elastic materials using an incremental crack growth procedure. The Visual Fortran programming language was used to develop the finite element code. The adaptive finite element mesh was generated using the advancing front method. Stress analysis for each increment was carried out using the adaptive mesh finite element technique. The equivalent stress intensity factor is the most essential parameter that should be accurately estimated for the mixed-mode loading condition which was used as the onset criterion for the crack growth. The node splitting and relaxation method advances the crack once the failure mechanism and crack direction have been determined. The displacement extrapolation technique (DET) was used to calculate stress intensity factors (SIFs) at each crack extension increment. Then, these SIFs were analyzed using the maximum circumferential stress theory (MCST) to predict the crack propagation trajectory and the fatigue life cycles using the Paris’ law model. Finally, the performance and capability of the developed program are shown in the application examples.

## 1. Introduction

In terms of structural integrity, two of the most significant issues are fatigue and fracture. Over the last years, this particular field of study has attracted a lot of attention, particularly regarding to the main failure procedures. An understanding of these mechanisms is essential to the process of developing components that are both more durable and more reliable. Computational design of structural components and materials with embedded cracks requires a comprehensive assessment of their reliability and a prediction of their remaining service life. The simulation of cracks growth has been conducted for various issues, including determining the fatigue life of a structure. This is often achieved by setting up a linear elastic fracture mechanics (LEFM) problem for each load step. Typically, in LEFM, the SIF of the crack is employed to simulate crack propagation by successive crack growth [1,2,3,4,5]. The stress intensity factors (SIFs) at the crack tip determine the fatigue crack growth rate according to linear elastic fracture mechanics (LEFM). To effectively predict the behavior of crack growth, a precise assessment of the SIFs at the crack tip are necessary, which has been expressed as a function of crack geometries and applied loads. Analytical SIFs solutions for idealized crack configuration and loading conditions existed in many handbooks and they could be applied to simple and regular structures [6,7,8]. However, in many structures the fatigue crack configurations are typically complicated and irregular, resulting in a variety of different failure modes. Therefore, analytical solutions will be inappropriate to predict the SIF solutions which can be estimated for these fatigue cracks using the results of finite element analysis. The two most common methods for the predictions of SIFs are the displacement extrapolation technique [9,10] and J-integral method [11,12]. Many numerical approaches have been developed over the years, including the finite element method (FEM), extended finite element method [13,14,15,16], method of Element Free Galerkin [17], discrete element method (DEM) [18,19,20], phase-field method [21], and cohesive element method [22,23]. In engineering applications, the finite element method is a sophisticated methodology for simulating complicated geometries and components. Two-dimensional crack propagation simulations were developed using many software tools and finite element packages with the aid of fracture mechanics, such as FRANC2D/L [24], ADAPCRACK2D [25,26], and ViDa [27]. The most time-consuming part of the analysis is characterizing the displacement and stresses fields, which is essential for estimating the SIFs. With the computing power available today, it is possible to perform structural analysis on large structure using conventional computers by using the developed program. For the modeling of two-dimensional fatigue crack growth using the LEFM assumptions, the development of this program was started in 2004 and since then, a wide range of features has been added with each new version [28,29,30,31,32,33,34,35,36]. Achieved results with the developed source code are comparable to those obtained with the available fracture mechanics’ commercial software. The robustness of the developed program was demonstrated in several scenarios, e.g., [28,29,30,31,32,33,34,35]. In terms of knowledge, using source code is appropriate for at least two reasons: first, understanding the basic algorithm that it uses, and second, acquiring programming skills in its development. This study also represents a scientific procedure that can be simply utilized by the researchers to use it as a guideline to construct their own program with the lowest amount of cost compared to commercial software.

## 2. Procedure of the Developed Program

**Figure 1:**
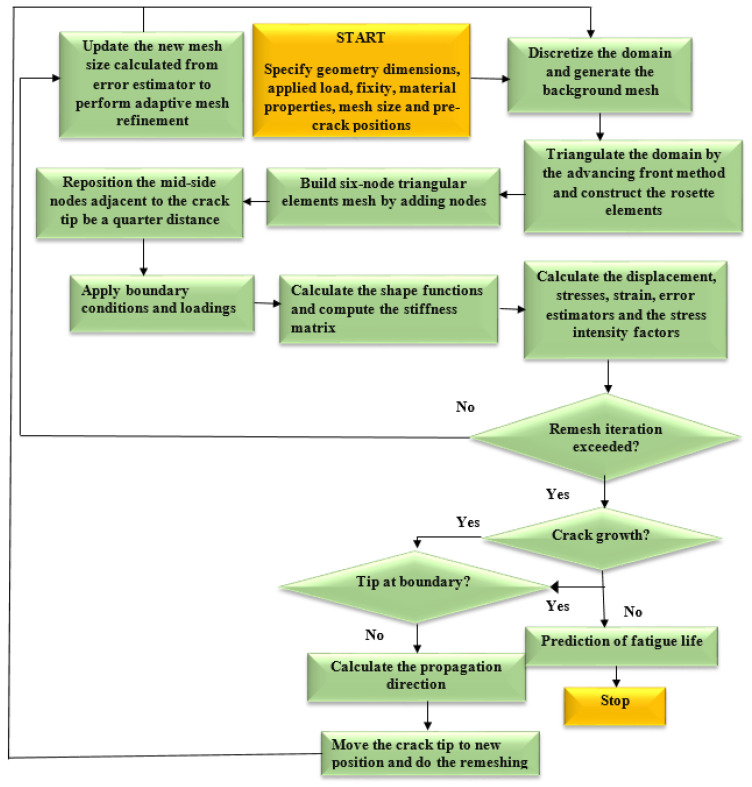
The program flowchart.

The 2D fatigue crack growth analysis began with specifying the geometrical dimensions, loads, material properties, and other constraints. During the pre-processing step, the finite element method is used to carry out an incremental stress analysis. At each stage of crack growth, the DET is used to determine the SIFs, that were then used by the MCST to predict the crack propagation trajectory and the fatigue life cycles are predicted using Paris’ law. Next, the advancing front method is implemented for generating the mesh which requires the description of the domain boundary, the generation of the elements, the mesh smoothing, and the renumbering of nodes [36]. The specific scale of every element, which is estimated by the error estimator, will be used to control mesh refinement. The solution components (stresses, displacement, strains, and so on) are transferred from the old mesh into the new mesh after the new mesh has been generated. In the next sections, a comprehensive explanation of the crack kinking criteria, background mesh creation, crack development increment, as well as node splitting and relaxation are provided. The computational scheme of the fatigue crack propagation program is shown in Figure 1.

### 2.1. Crack Kinking Criteria

Two crucial factors are taken into consideration at every incremental crack growth step. First, determine whether the crack propagates and, if so, in what direction. Second, two criteria should be used based on two conditions: one for crack kinking and another for crack propagation. The criterion for crack growth is either the conventional energy approach or by the stress intensity approach. According to the conventional energy approach, a crack grows whenever the energy release rate reaches a significance value of the material’s fracture toughness [37]. On the other hand, according to the stress intensity approach, a crack grows whenever the stress intensity factor at the crack tip exceeds the material’s fracture toughness in the case of static loading or the equivalent stress intensity factor exceeds the threshold stress intensity factor in the case of fatigue loading. The maximum circumferential stress theory was used in this study to calculate the crack direction angle [38]. According to this theory, when isotropic materials are subjected to mixed mode loading, the crack grows in a normal direction to the direction in which the tangential tensile stress is maximum. The following expressions provide the formulas for calculating the tangential stresses in polar coordinates for the two modes of SIFs, *K_I_*, and *K_II_* [38,39]:

(1) $$\begin{array}{l} \sigma_{r}\;=\;\frac{1}{\sqrt{2\pi r}}\cos(\theta /2)\left(K_{I}[1+\sin^{2}(\theta /2)]+\frac{3}{2}K_{II}\sin\theta -2K_{II}\tan(\theta /2)\right)\\ \sigma_{\theta }=\frac{1}{\sqrt{2\pi r}}\cos(\theta /2)\left[K_{I}\cos^{2}(\theta /2)-\frac{3}{2}K_{II}\sin\theta \right]\\ \tau_{r\theta }=\frac{1}{\sqrt{2\pi r}}\frac{\cos(\theta /2)}{2}\left[K_{I}\sin\theta +K_{II}(3\cos\theta -1)\right]\;\;\;\;\;\;\;\; \end{array}$$

where $\sigma_{r}$ represents the radial component of normal stress, $\sigma_{\theta }$ represents the tangential component of normal stress, and $\tau_{r\theta }$ represents the shear stress component. When solving $d\sigma_{\theta }/d\theta =0$ for θ, the solution is given as:

(2) $$K_{I}\sin\theta +K_{II}\left(3\cos\theta -1\right)=0$$

From which the kinking angle can be obtained as:

(3) $$\theta =\cos^{-1}\;\left(\frac{3K_{II}^{2}+K_{I}\sqrt{K_{I}^{2}+8K_{II}^{2}}}{K_{I}^{2}+9K_{II}^{2}}\right)$$

The sign of θ must be opposite to the sign of *K_II_* to ensure the maximum stress associated with the crack increment [40].

### 2.2. The Background Mesh Generation

**Figure 2:**
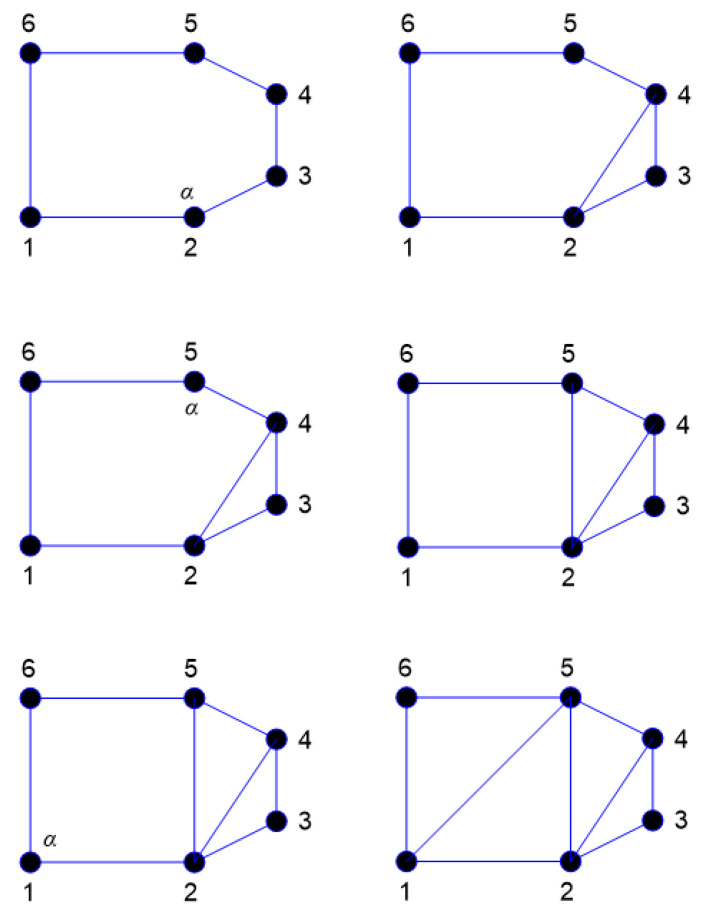
Division of a polygon based on the proposed dichotomy method.

**Figure 3:**
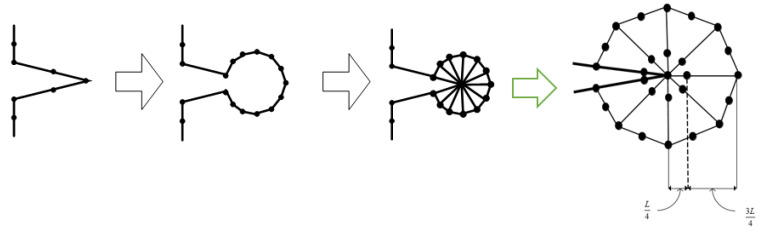
Steps of generating quarter-point elements around a crack tip.

**Figure 4:**
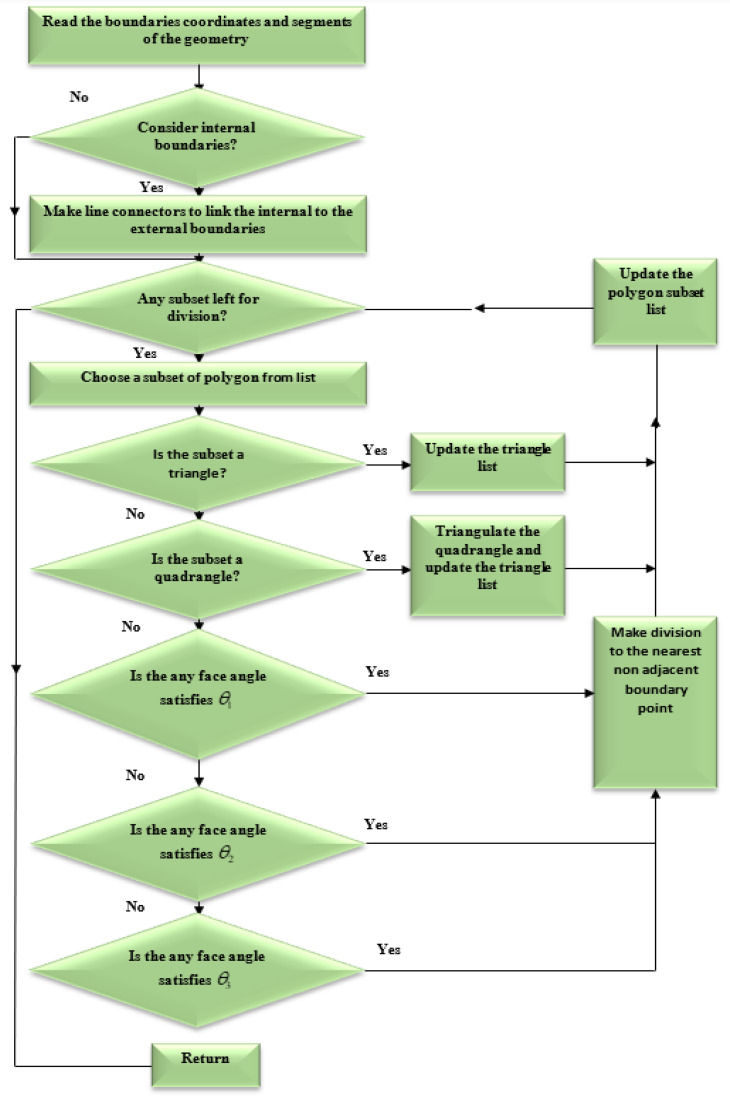
Flowchart of the background mesh generation.

An appropriate technique must be used during each load step to ensure that the generated background mesh covers the whole computational domain. The background mesh is generated by using the dichotomy approach, which uses all of the original boundary nodes or mainly the external boundary nodes of the shape to build the background mesh triangular. In this method, the computing area was modeled as a polygon. The boundary triangulations were acquired by splitting and repeatedly dividing the computational area into two sub-sets till the simplest polygon sub-sets were generated. Consequently, any internal boundaries, such as holes, must be connected to the external boundary by connecting lines. The internal boundaries would be compelled to be a part of the continuous line of the external boundaries, resulting in a polygonal computational domain. The clockwise direction is used to set the orientation of internal borders, whereas the counterclockwise direction is used to set the orientation direction of external boundaries. The shortest path between internal and external boundary points is used to construct the connector line [41]. The proposed method is shown in Figure 2, which begins by separating each initial identified boundaries points with a large face angle and producing an angle range for determining the closest nonadjacent point to be connected with a division line. The angle range has been selected in order to facilitate the generation of high quality polygon subset forms by the division. In the event that the search for a point not neighboring is unsuccessful, the division will begin at a position on the border that has a smaller face angle. In accordance with the precedence, the identification for the face angle size is as follows: $\pi \leq \theta_{1}<2\pi ,\;\;\;\;\;\pi /2<\theta_{2}<\pi ,\;0<\theta_{3}\leq \pi /2.$ As a result, in order to precisely represent the field singularity near the crack tip, singular elements must be constructed. As the advancing front technique creates triangle elements beginning from the boundaries faces, the region surrounding the crack tip is must to be separated before constructing of the singular elements [36]. This area is separated by creating nodes in the rosette form surrounding the crack tip and extracting the node at the crack tip and the connected boundaries segments. By introducing new boundary segments connecting the new nodes, the template region is temporarily “cut off” from the initial domain followed by the triangulation of the whole domain using the advancing front method. The triangles of the rosette elements are then formed, as shown in Figure 3. Finally, a mid-side is added to each triangle edge to create six node triangles, with the exception of the rosette components, where the mid side nodes for the triangle edges related to the crack tip are shifted a quarter of the edges length closer to the tip of the crack. The flow chart for generating the background mesh is shown in Figure 4.

### 2.3. Crack Growth Increment

The ratio of the two modes of SIFs (*K_II_/K_I_*) is indirectly proportional to the crack growth increment (∆*a*), which was chosen to be 5–10 percent of the initial crack length which was more appropriate for the smoothed crack propagation curvature trajectory. When *K_II_* is comparatively larger than *K_I_*, it implies a mixed-mode relation. Consequently, a shorter incremental size is necessary in order to adequately justify the smooth crack propagation trajectory. It was observed that the length of crack-extension increments had no effect on the results of stress intensity factors for increments that were less than 5% of the initial crack length [42].

This percentage range, however, may be changed as appropriate, as several previous studies utilizing a 20–50 percent range [38,43,44]. As a direct consequence of this, the Lagrange interpolation provides an approximation of the incremental crack growth as follows:

(4) $$\Delta a=\left(\left(1-\left|\frac{K_{II}}{K_{I}}\right|\right)\left(20\%\right)+\left|\frac{K_{II}}{K_{I}}\right|\left(5\%\right)\right)a$$

where *K_I_* and *K_II_* are the first and second modes of SIFs.

### 2.4. Node Splitting and Relaxation

**Figure 5:**
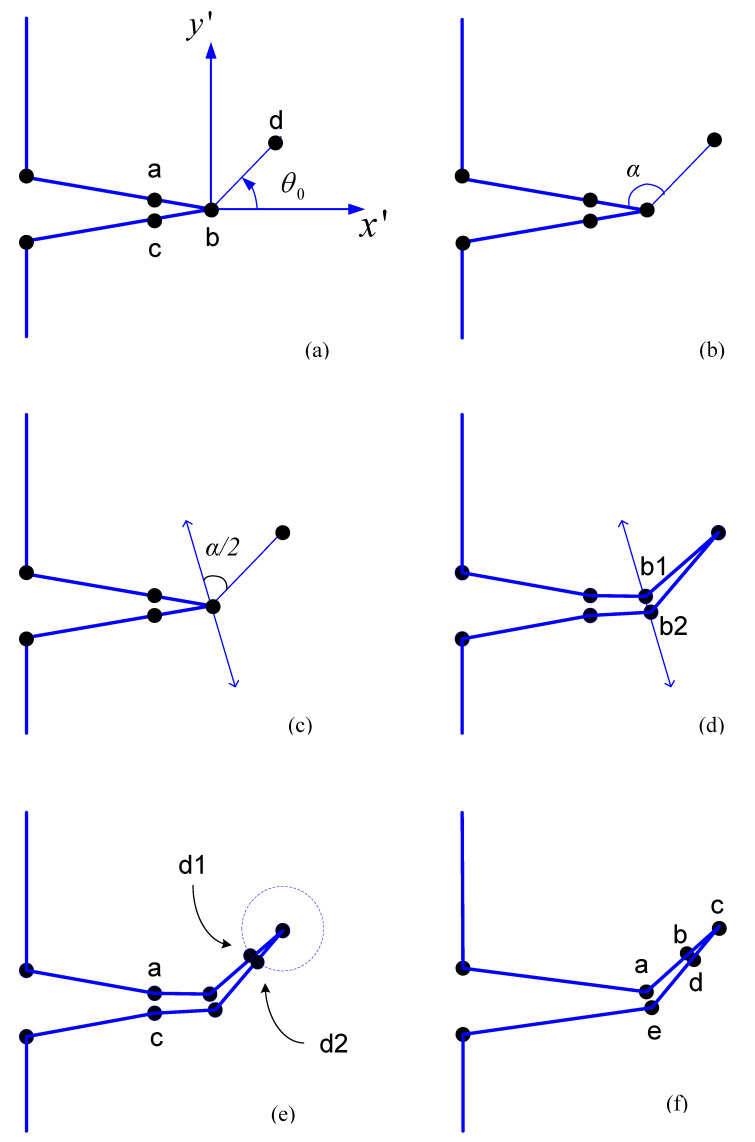
Procedure for splitting a crack node and updating boundary nodes. (**a**) step 1 (**b**) step 2 (**c**) step 3 (**d**) step 4 (**e**) step 5 (**f**) step 6.

Relaxation of the split nodes is the release of the nodes in accordance with their mechanical properties. When the criterion for crack propagation are satisfied at a particular crack tip, the crack tip node has to be split into two separate nodes so that the crack opening can be simulated. If it is necessary to display the deformation, the displacement should be continually updated using the coordinates of the boundary nodes. The splitting direction is determined by dividing the angle between the segment that initially includes the present crack-tip and the segment that connects the present crack-tip to the predicted next crack-tip, upwards and downwards. Figure 5 shows the node splitting and relaxing procedure. Assuming that *a* and *c* are the initial nodes that come before and after the crack tip *b*, respectively, and that *d* is the estimated next crack tip. The incrementally crack length will be |*bd*|, and the trajectory will be as indicated in Figure 5a. As illustrated in Figure 5b, the angle α between segments ab and bd is divided and the splitting direction is chosen at α/2 as shown in Figure 5c, which involves both upwards and downwards directions. If the length of the splitting is set to Δs, the length of each splitting node b1 and b2 from the original crack tip is Δs/2 as shown in Figure 5d. The new segments connecting the new crack tip to the split nodes should be the same length. If they are not, it will be ridiculous to create the uniform rosette template later. As a result, two additional boundary nodes must be inserted, as seen in Figure 5e. Finally, only a total of three nodes need to be added in every step of the crack growth as shown in Figure 5f, and the geometry can now be updated.

In order to restart the process from the beginning, the constraint, loading, and crack tip data must all be updated to reflect the inclusion of new boundary segments.

### 2.5. Refinement of the Adaptive Mesh

Refining the adaptive mesh is an optimization approach used in the field of finite element mesh. Refinement of meshes along the crack and towards its tip is initially achieved using a customized adaptive mesh refinement. This approach is based on an a posteriori error estimate derived from the previous mesh generation. The relative stress norm error is the metric that is used to provide a reasonable approximation of the error in the mesh refinement. The ratio of the standard stress error of the elements to the average standard stress error of the whole area was determined by using the adaptive mesh optimization of the *h*-type. In this manner, the mesh size of each element is represented as follows:

(5) $$h_{e}=\sqrt{2A_{e}}$$

where *A_e_* is the area of the triangular element. The representation of the average norm stress error throughout the entire domain is expressed as:

(6) $$\begin{array}{l} {\left\|\hat{e}\right\|}^{2}=\frac{1}{m}\sum_{e=1}^{m}\int_{\Omega^{e}}\sigma^{T}\sigma d\Omega \\ \;\;\;\;\;\;\;=\frac{1}{m}\sum_{e=1}^{m}\int_{\Omega^{e}}{\left\{\begin{array}{c} \sigma_{x}\\ \sigma_{y}\\ \tau_{xy}\\ \sigma_{z} \end{array}\right\}}^{T}\left\{\begin{array}{c} \sigma_{x}\\ \sigma_{y}\\ \tau_{xy}\\ \sigma_{z} \end{array}\right\}d\Omega \end{array}$$

where *m* denotes the total number of domain elements. In the finite element method, the integration with the triangular isoparametric domain is converted by the summation of quadratics in accordance with the Radau principle as following:

(7) $$\begin{array}{ll} ∥e{∥}_{e}^{2} & =\overset{1}{\underset{-1}{\int }}\overset{1}{\underset{1}{\int }}{\left(\left\{\begin{array}{c} \sigma {\left(\xi ,\eta \right)}_{x}\\ \sigma {\left(\xi ,\eta \right)}_{y}\\ \tau {\left(\xi ,\eta \right)}_{xy}\\ \sigma {\left(\xi ,\eta \right)}_{z} \end{array}\right\}-\left\{\begin{array}{c} \sigma {\left(\xi ,\eta \right)}_{x}^{*}\\ \sigma {\left(\xi ,\eta \right)}_{y}^{*}\\ \tau {\left(\xi ,\eta \right)}_{xy}^{*}\\ \sigma {\left(\xi ,\eta \right)}_{z}^{*} \end{array}\right\}\right)}^{T}\left(\left\{\begin{array}{c} \sigma {\left(\xi ,\eta \right)}_{x}\\ \sigma {\left(\xi ,\eta \right)}_{y}\\ \tau {\left(\xi ,\eta \right)}_{xy}\\ \sigma {\left(\xi ,\eta \right)}_{z} \end{array}\right\}-\left\{\begin{array}{c} \sigma {\left(\xi ,\eta \right)}_{x}^{*}\\ \sigma {\left(\xi ,\eta \right)}_{y}^{*}\\ \tau {\left(\xi ,\eta \right)}_{xy}^{*}\\ \sigma {\left(\xi ,\eta \right)}_{z}^{*} \end{array}\right\}\right)t^{e}\det J^{e}d\xi d\eta \\ & =\overset{3}{\underset{p=1}{\sum }}{\left(\left\{\begin{array}{c} \sigma {\left(\xi_{p},\eta_{p}\right)}_{x}\\ \sigma {\left(\xi_{p},\eta_{p}\right)}_{y}\\ \tau {\left(\xi_{p},\eta_{p}\right)}_{xy}\\ \sigma {\left(\xi_{p},\eta_{p}\right)}_{z} \end{array}\right\}-\left\{\begin{array}{c} \sigma {\left(\xi_{p},\eta_{p}\right)}_{x}^{*}\\ \sigma {\left(\xi_{p},\eta_{p}\right)}_{y}^{*}\\ \tau {\left(\xi_{p},\eta_{p}\right)}_{xy}^{*}\\ \sigma {\left(\xi_{p},\eta_{p}\right)}_{z}^{*} \end{array}\right\}\right)}^{T}\left(\left\{\begin{array}{c} \sigma {\left(\xi_{p},\eta_{p}\right)}_{x}\\ \sigma {\left(\xi_{p},\eta_{p}\right)}_{y}\\ \tau {\left(\xi_{p},\eta_{p}\right)}_{xy}\\ \sigma {\left(\xi_{p},\eta_{p}\right)}_{z} \end{array}\right\}-\left\{\begin{array}{c} \sigma {\left(\xi_{p},\eta_{p}\right)}_{x}^{*}\\ \sigma {\left(\xi_{p},\eta_{p}\right)}_{y}^{*}\\ \tau {\left(,\eta_{p}\right)}_{xy}^{*}\\ \sigma {\left(\xi_{p},\eta_{p}\right)}_{z}^{*} \end{array}\right\}\right)t^{e}\det J^{e}W_{p} \end{array}$$

and similarly

(8) $$∥\hat{e}{∥}^{2}=\frac{1}{m}\overset{m}{\underset{e=1}{\sum }}\overset{3}{\underset{p=1}{\sum }}{\left(\left\{\begin{array}{c} \sigma {\left(\xi_{p},\eta_{p}\right)}_{x}\\ \sigma {\left(\xi_{p},\eta_{p}\right)}_{y}\\ \tau {\left(\xi_{p},\eta_{p}\right)}_{xy}\\ \sigma {\left(\xi_{p},\eta_{p}\right)}_{z} \end{array}\right\}\right)}^{T}\left(\left\{\begin{array}{c} \sigma {\left(\xi_{p},\eta_{p}\right)}_{x}\\ \sigma {\left(\xi_{p},\eta_{p}\right)}_{y}\\ \tau {\left(\xi_{p},\eta_{p}\right)}_{xy}\\ \sigma {\left(\xi_{p},\eta_{p}\right)}_{z} \end{array}\right\}\right)t^{e}\det J^{e}W_{p}$$

where *t^e^* is the element thickness for a plane stress and *t^e^* =1 for a plane strain. *W_P_* is a weighting factor, and $J^{e}$ is the Jacobian matrix, which is represented as:

(9) $$J^{e}\;=\;\left[\begin{array}{cc} \frac{\partial x}{\partial \xi } & \frac{\partial y}{\partial \xi }\\ \frac{\partial x}{\partial \eta } & \frac{\partial y}{\partial \eta } \end{array}\right]\;=\;\left[\begin{array}{cc} \sum_{i=1}^{r}\frac{\partial N_{i}^{e}}{\partial \xi }\;x_{i}^{e} & \sum_{i=1}^{r}\frac{\partial N_{i}^{e}}{\partial \xi }\;y_{i}^{e}\\ \sum_{i=1}^{r}\frac{\partial N_{i}^{e}}{\partial \eta }\;x_{i}^{e} & \sum_{i=1}^{r}\frac{\partial N_{i}^{e}}{\partial \eta }\;y_{i}^{e} \end{array}\right]$$

As a consequence, the relative stress norm error $\zeta_{e}$ for each element is much less than 5%, which is an acceptable range for a broad range of engineering applications [36]. Hence,

(10) $$\zeta_{e}=\frac{{\left\|e\right\|}_{e}}{\left\|\hat{e}\right\|}\leq \zeta$$

and the permissible error level for the new element is defined as follows:

(11) $$\varepsilon_{e}=\frac{{\left\|e\right\|}_{e}}{\zeta \left\|\hat{e}\right\|}\leq 1$$

It indicates that each individual element with $\varepsilon_{e}>1$ must undergo further refinement, and the new mesh size must be expected. In this instance, the asymptotic convergence rate criterion are applied, which assumes the following:

(12) $${\left\|e\right\|}_{e}\propto h_{e}^{p}$$

where *p* is the approximation of the polynomial order. For the quadratic polynomial, the new element size is estimated as:

(13) $$h_{N}=\frac{1}{\sqrt{\varepsilon_{e}}}h_{e}$$

The old mesh will be used as the new background mesh and the advancing front method is repeated, depending on the user-specified number of mesh refinements.

### 2.6. Displacement Extrapolation Technique

**Figure 6:**
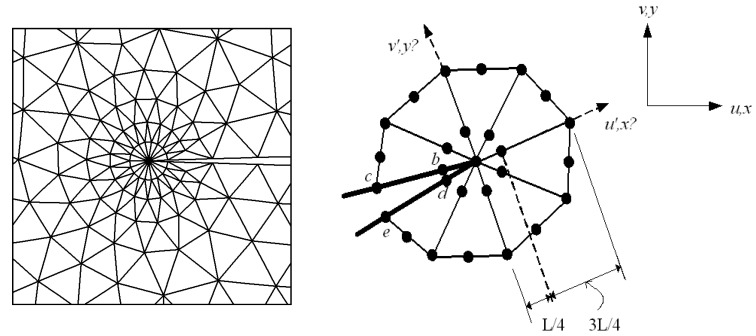
Triangular rosette elements and coordinates surrounding the crack tip.

The displacement extrapolation technique is used for linear elastic materials to calculate the SIFs from finite element nodal displacement. In this method, the crack extremity is totally surrounded by triangular quarter-point singular elements, while the remaining areas are covered by six-node isoparametric elements. The displacement component of the partial nodes located around the tip and along the crack line is calculated during this extrapolation. The required formulas are used for this component in order to obtain the stress intensity factors that correspond to these nodes. Figure 6 shows the detailed rosette triangle elements formed around the crack tip using the displacement extrapolation technique.

The following formulae were used to compute the SIFs [43]:

(14) $$K_{I}=\frac{E}{3(1+\nu )(1+\kappa )}\sqrt{\frac{2\pi }{L}}\left[4(v_{b}^{\prime }-v_{d}^{\prime })-\frac{\left(v_{c}^{\prime }-v_{e}^{\prime }\right)}{2}\right]$$

(15) $$K_{II}=\frac{E}{3(1+\nu )(1+\kappa )}\sqrt{\frac{2\pi }{L}}\left[4(u_{b}^{\prime }-u_{d}^{\prime })-\frac{\left(u_{c}^{\prime }-u_{e}^{\prime }\right)}{2}\right]$$

where *E* is the elastic modulus, ν is the Poisson’s ratio, κ is the elastic factor represented as:

(16) $$\kappa \;=\;\left\{\begin{array}{c} 3-4\nu \;for\;plane\;strain\;\;\\ \frac{\left(3-\nu \right)}{\left(1+\nu \right)}\;for\;plane\;stress\;\;\; \end{array}\right.$$

and *L* denotes the length of the quarter-point element. Where ${u^{\prime }}_{}$ and ${v^{\prime }}_{}$ are the displacement components in the x′ and y′ directions, respectively, as denoted in Figure 5.

## 3. Numerical Results and Discussion

### 3.1. Single Edge Notched Specimen under Shear Load

**Figure 7:**
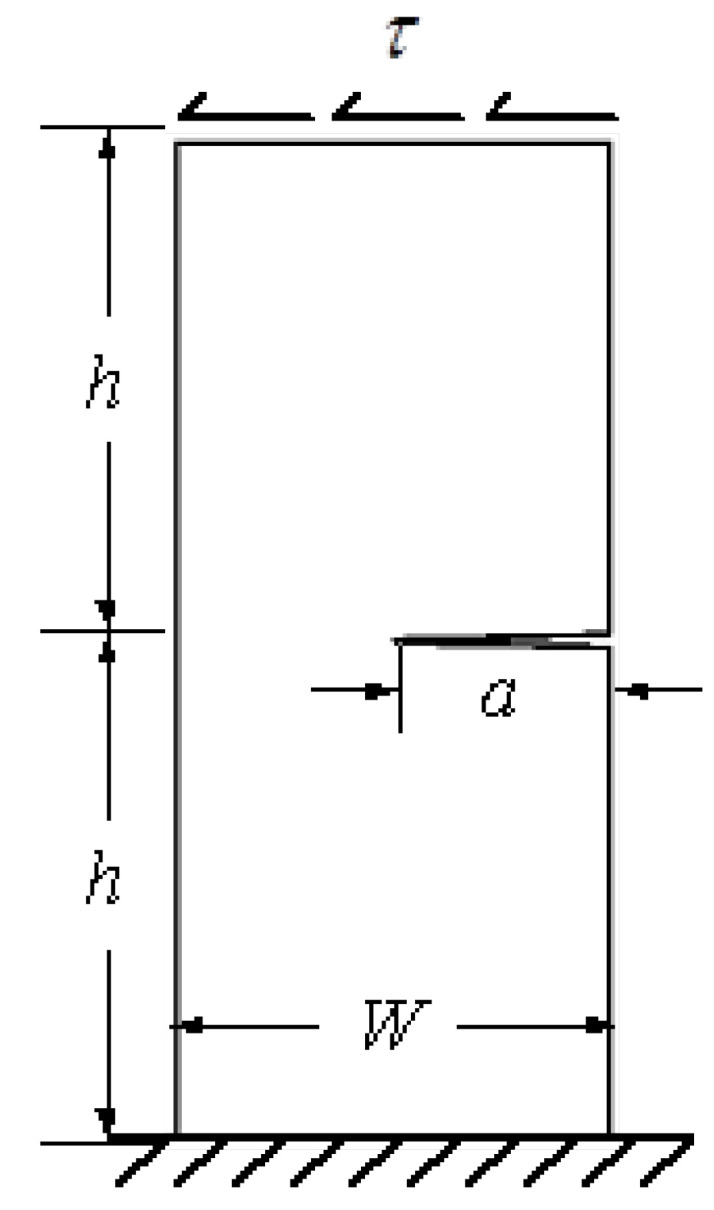
Problem statement for the single edge notched specimen under shear load.

**Figure 8:**
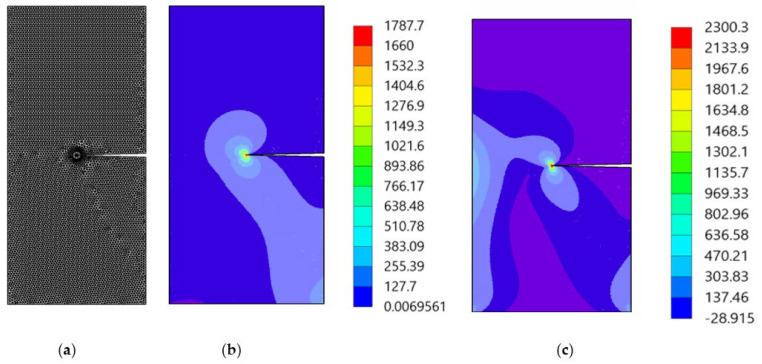
(**a**) The adaptive mesh, (**b**) maximum principal stress (MPa), and (**c**) von Mises stress (MPa).

As shown schematically in Figure 7, the growth of an edge crack in a rectangular plate subjected to a shear stress, τ = 1 unit is considered. The initial crack length is a = 3.5 cm, the width of the plate is *W* = 7 cm, and the height is 2*h* = 16 cm. The material properties are selected as modulus of elasticity, *E* = 30 × 10^6^ N/mm^2^ and the Poisson’s ratio, *ν* = 0.25. It is assumed that the plane strain condition is applied in this case. Figure 8 displays the initial adaptive mesh as well as the contour distribution of the maximum principal stress and von Mises stress for the first step before crack growth. The highest values of maximum principal stress and von Mises stress are visible at the tip of the crack.

**Figure 9:**
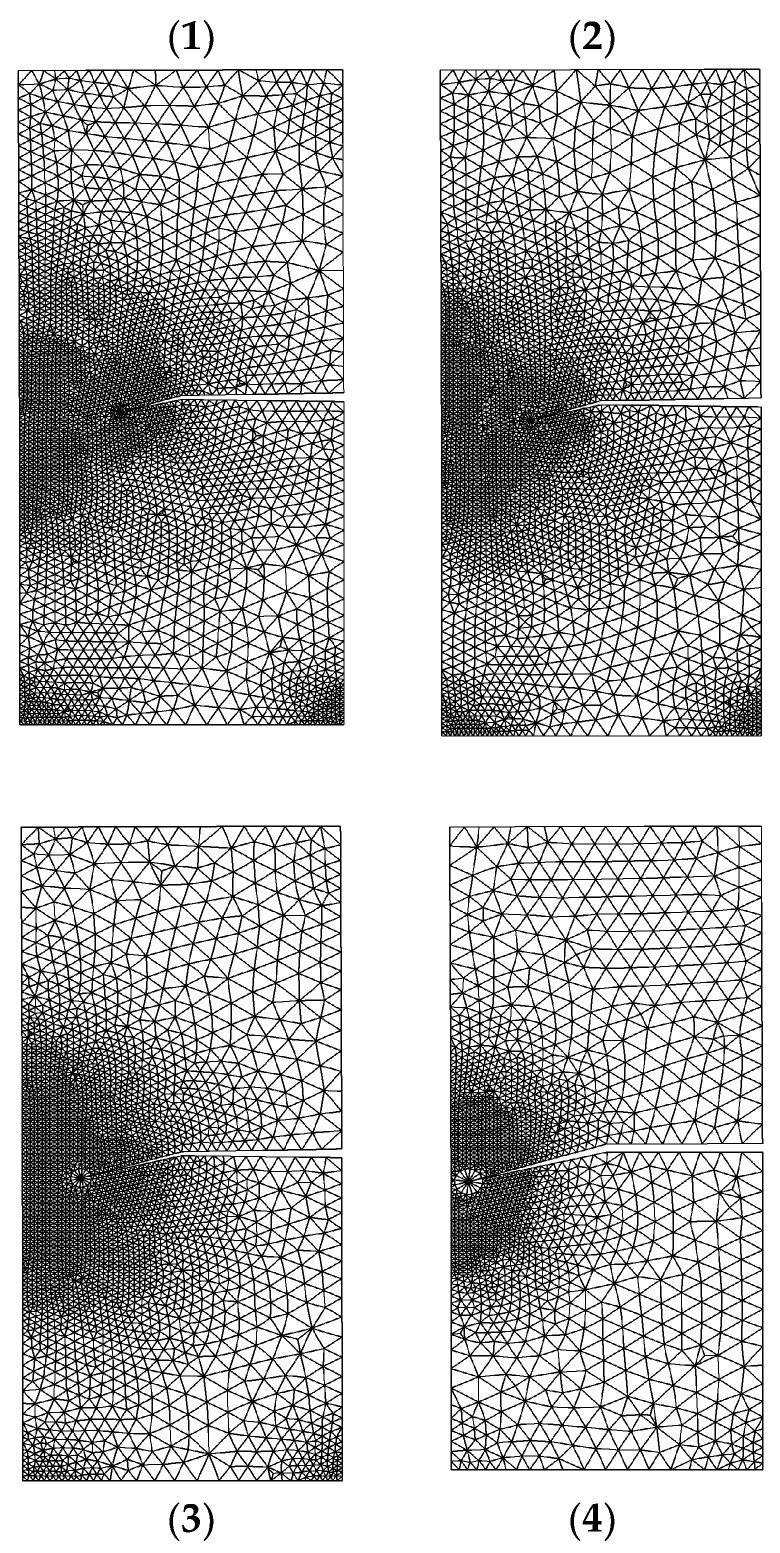
The crack growth trajectory for a single edge cracked plate under shear loading.

Figure 9 depicts the crack growth trajectory in four selected steps, each representing an adaptive mesh distribution. The adaptive mesh refinement initially occurs in local area near crack front. As the number of adaptive local refinements increases, the refinement domain shrinks to a smaller area in the surrounding area of the crack tip. At the beginning of crack growth, the mode II stress intensity factor dominated the crack direction. However, as the crack trajectory proceeds, the magnitude of the mode I stress intensity factor becomes significantly higher, as demonstrated by the straight crack path at the end of the crack trajectory.

**Figure 10:**
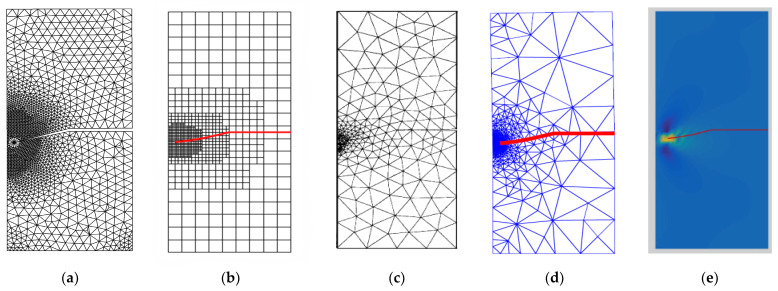
Predicted crack growth path, (**a**) present study, (**b**) numerical results [46], (**c**) numerical results [43] (**d**) numerical results [45], (**e**) numerical results [47].

**Figure 11:**
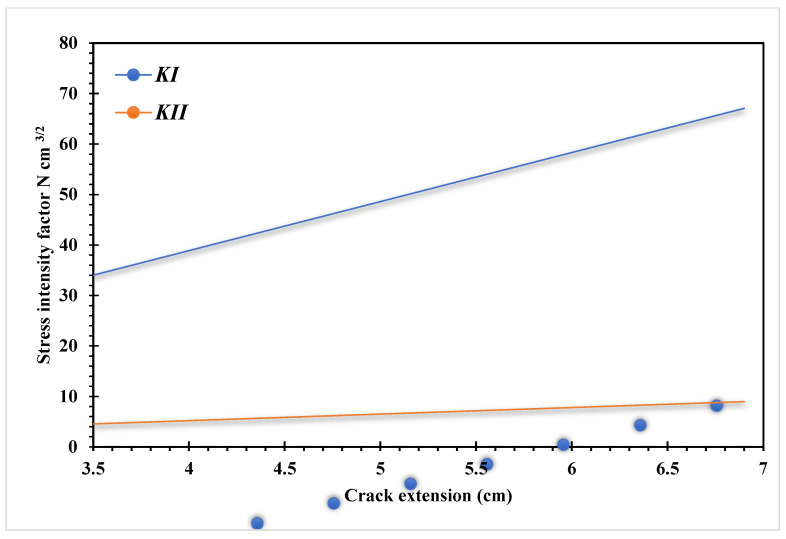
Stress intensity factors for each step of crack growth.

**Figure 12:**
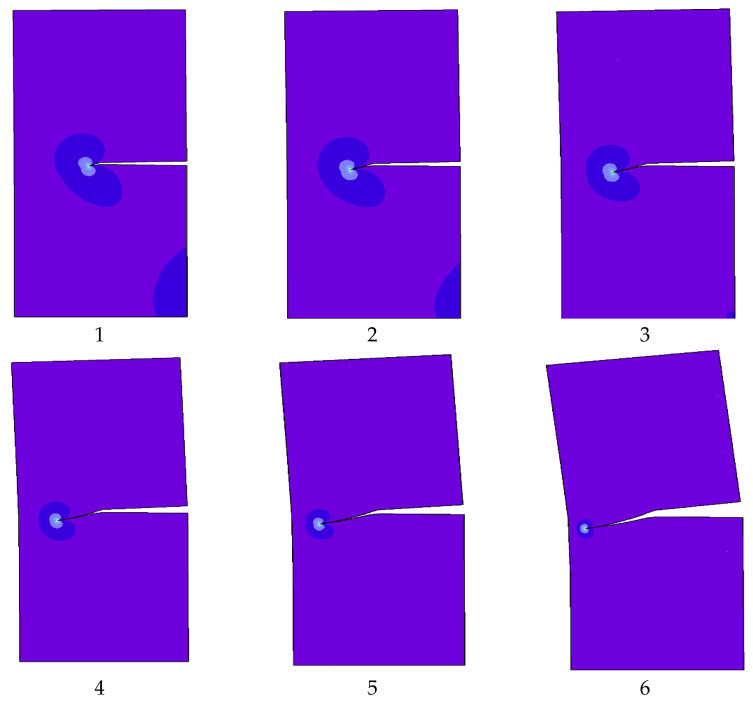
Steps of crack propagation simulation for the single edge notched specimen under shear load.

The predicted stress intensity factors were $K_{I}=34.00\;N\;{\mathrm{cm}}^{-3/2}$ and $K_{II}=4.55\;N\;{\mathrm{cm}}^{-3/2}$ that were compared to the reference values [43,45,46] of 34.00 and 4.55, 34.1 and 4.52, and 34.00 and 4.55 respectively. The predicted crack propagation trajectory in this study matches the crack growth trajectory obtained in [42] using a singular edge-based smoothed finite element method. Moreover, the predicted crack growth path obtained by [43] using FEM and the adaptive Delaunay triangulation, the predicted crack growth path obtained by [46] using adaptive extended isogeometric analysis (XIGA) based on locally refined B-splits, as well as the predicted crack growth path obtained by [47] using extended element-free Galerkin method are shown in Figure 10a–e respectively. Figure 11 shows the predicted values of stress intensity factors for each step of crack growth. The overall steps of crack propagation are depicted in Figure 12 as a contour of the maximum principal stress.

### 3.2. Modified Four-Point Bending Beam

**Figure 13:**
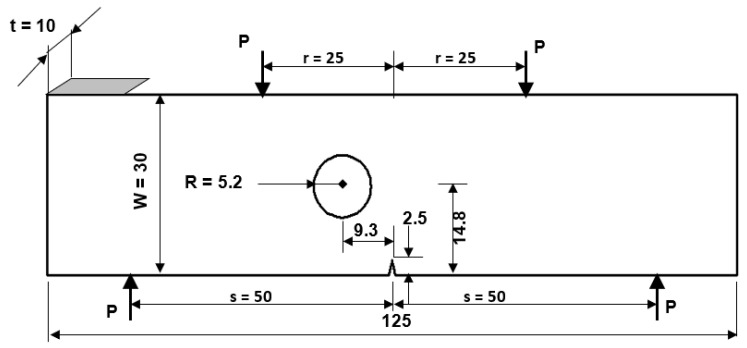
Geometrical dimensions and load positions of the modified four-point bending beam (mm).

**Figure 14:**
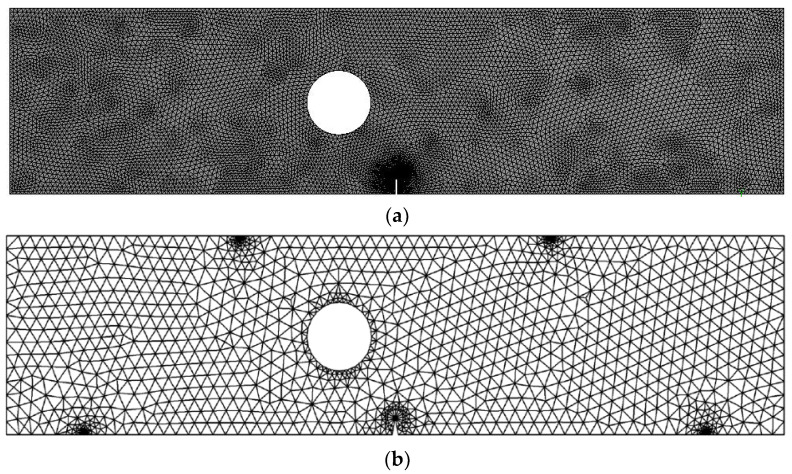
Adaptive mesh for the modified four-point bending beam, (**a**) dens mesh and (**b**) coarse mesh.

Figure 13 depicts a modified four-point bending specimen with a single crack and one hole located 9.3 mm from the left side of the crack center. This specimen was simulated under fatigue loading with constant amplitude load ratio R = 0.1, and the amount of the applied loads are *P* = 100 N. The initial mesh of this geometry is displayed in Figure 14 with two types of mesh density. The material properties are elastic modulus, *E* = 205 GPa, Poisson’s ratio, υ = 0.333, yield strength, $\sigma_{y}$ = 491 MPa, threshold stress intensity factor, *K_th_ =* $11.6\;\mathrm{MPa}\sqrt{m}$, Paris’ law coefficient, $C=4.5\times 10^{-10}$ and Paris law exponent, *m* = 2.1.

**Figure 15:**
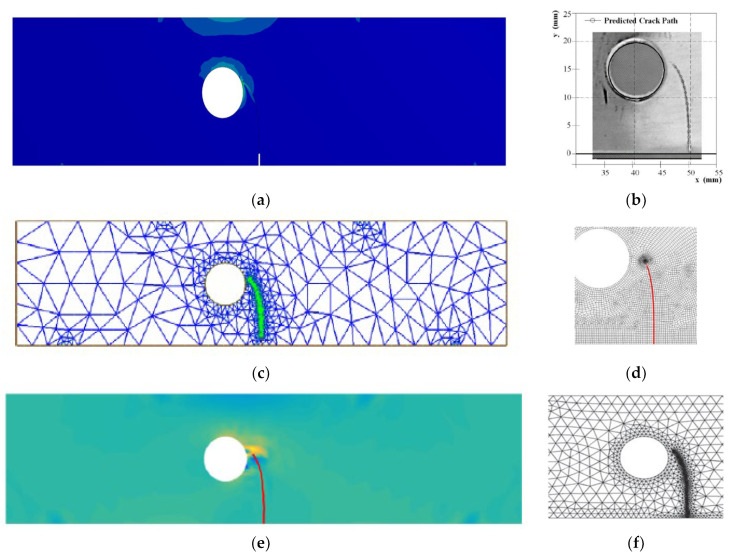
A comparison of the four point bending beam’s crack growth path, (**a**) present study, (**b**) experimental results [48], (**c**) numerical [49], (**d**) numerical [50], (**e**) numerical [47], (**f**) numerical [27].

**Figure 16:**
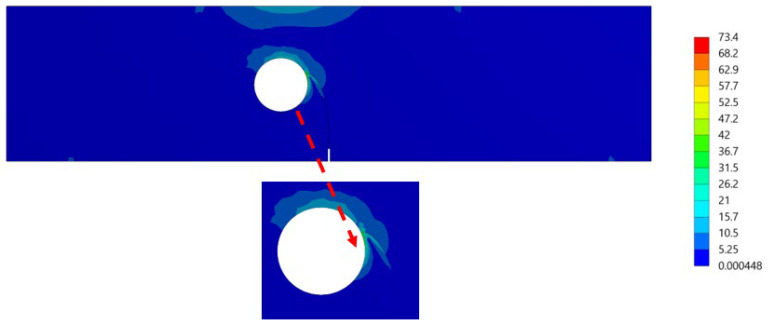
The distribution of a von Mises stress (MPa) at the last step of the crack’s propagation.

**Figure 17:**
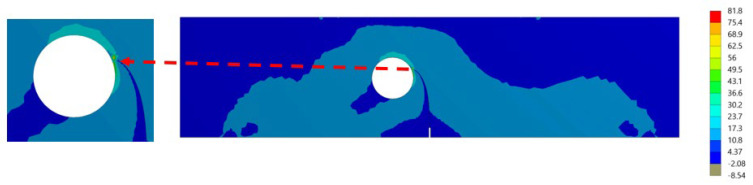
The distribution of the maximum principal stress (MPa) at the last step of the crack’s propagation.

It was found that the estimated crack growth path agreed the experimental path observed by [48]. In addition, the predicted crack propagation direction agrees with the predicted trajectories of other previous numerical [49,50], and [47]. The study in [49] utilizes FEM based on local Lepp–Delaunay meshes refinement, the work [50] uses FEM with configurational forces, and the method in [47] applies coupled extended mesh free–smoothed mesh free method. The results were compared to the finite element results obtained in [27] uses ViDa program as shown in Figure 15a–f, respectively. It is important to observe, in the early stages of the expansion of the crack, that the crack expands in a straight path. That is because the crack tip is still relatively distant from the hole. However, since the hole influences the direction in which the crack grows, the direction in which the crack propagates changes at a significant angle and progressively moves closer to the hole. Figure 16 depicts the von Mises stress, together with an enlarged view of the region around the crack tip. Figure 17 illustrates the distribution of the maximum principal stress distribution, with an enlarged view of the region around the crack tip.

As can be seen in the Figure 17, at the beginning of the crack growth process, the crack started to grow in a straight line while the first mode of SIFs controlled the crack propagation path. The second mode of stress intensity components increased in value as the crack grew toward the hole and altered its direction when the hole’s existence influenced the crack’s direction.

When determining the crack propagation rate and the fatigue life of a component, the SIFs are the most critical factors to be considered. Following is the analytical solution of the normalized stress intensity factor solution for the regular four-point bending beam that does not include a hole [51]:

(17) $$f(a/W)=K_{I}/\frac{6P(s-r)\sqrt{\pi a}}{W^{2}t}$$

where *K_I_* represents the first mode of SIFs, *f(a/W)* refers to the normalized SIF, *W* is the specimen width, *t* is specimen thickness, *P* is the applied load, *s* and *r* are the distances represented in Figure 1, and *a* is the crack length.

**Figure 18:**
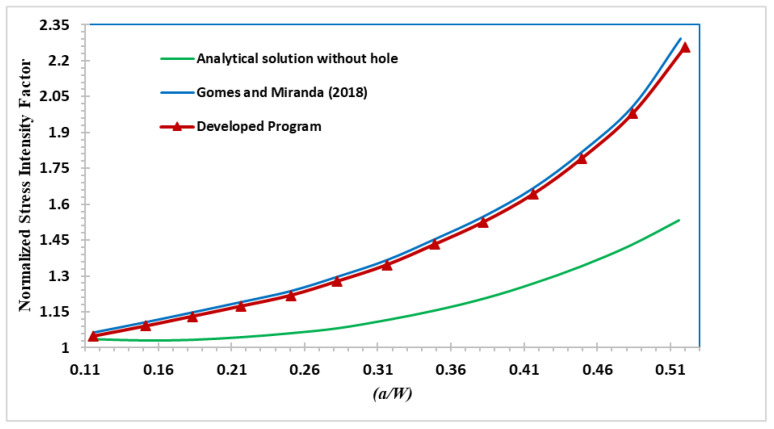
Normalized SIFs for the regular and modified four-point bending beam compared to Gomes and Miranda [27].

To demonstrate the influence of the presence of a hole on the crack propagation direction, which is correlated to the associated SIFs, the predicted values of the normalized SIF were compared to the calculated values from the analytical solution represented in Equation (17). Additionally, the dimensionless SIF values were calculated by [27] using the boundary element method (BEM) with BemCracker2D (BC2D) program, as depicted in Figure 18. It was noticed that the hole’s insertion significantly influences the normalized stress intensity factor values.

**Figure 19:**
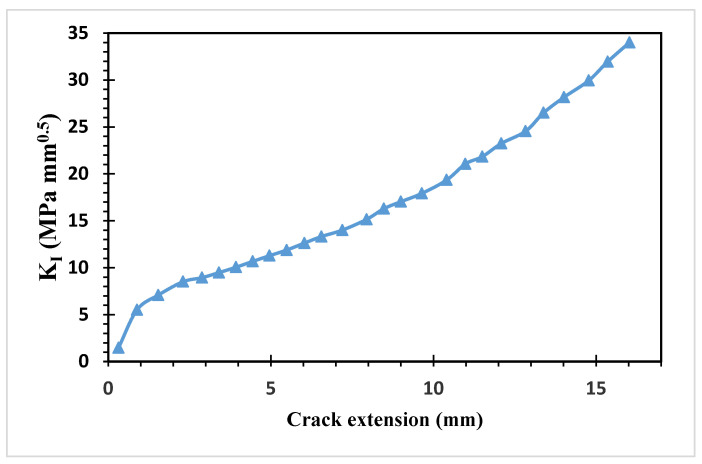
First mode of stress intensity factors.

**Figure 20:**
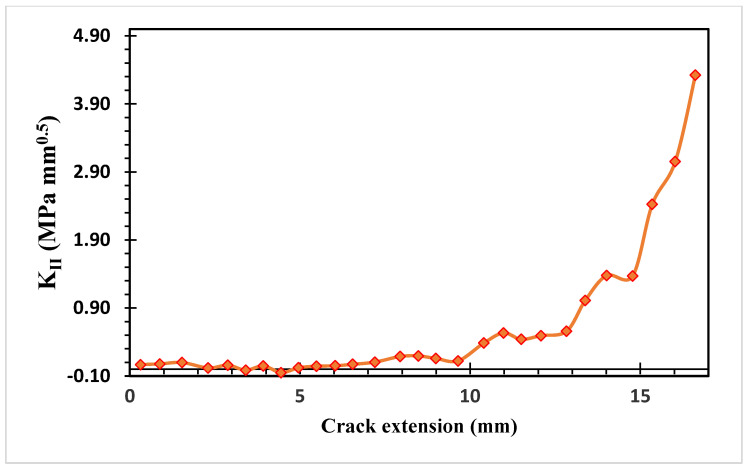
Second mode of stress intensity factors.

Figure 19 and Figure 20 show the estimated values of both modes of SIFs. At the beginning of the crack’s propagation, *K_I_* dominated the crack’s direction because *K_II_* values were smaller than to *K_I_* values. *K_II_* was then steadily raised to a maximum value of 4.323 MPa(mm)^1/2^ as the second mode of stress intensity factors, resulting in a change in the trajectory of the crack to grow toward the hole.

**Figure 21:**
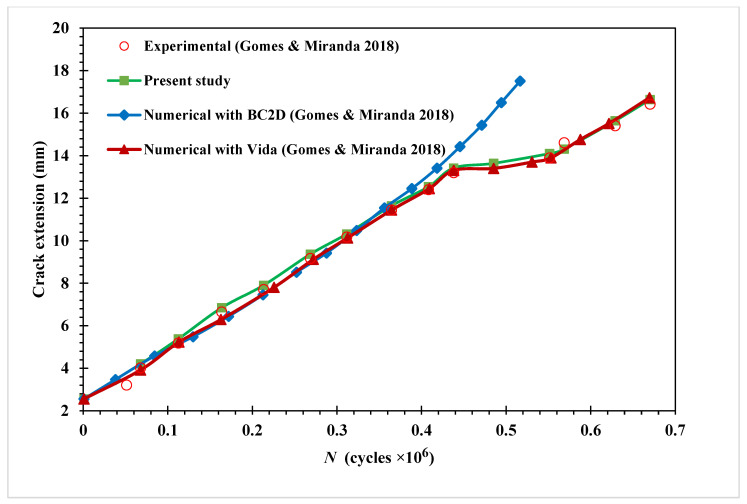
Modified four-point bending beam fatigue life comparison with [27].

As shown in Figure 21, the calculated fatigue life cycles are compared to the experimental data obtained by [27], and the numerical results obtained by the same authors using two different software: Vida and BemCracker2D (BC2D). While ViDa and BemCracker2D are two-dimensional crack growth programs based on the finite element method and the dual boundary element method. In contrast to the BemCracker2D findings, which deviated from the experimental data in the last stages of crack growth, this figure shows that the present study’s results were in line with both the experimental data and the numerical results produced by Vida 98 software.

## 4. Conclusions

In this study, a fatigue crack propagation methodology based on the adaptive FEM applies to analyze fractures with mixed-mode of crack growth behavior. The displacement extrapolation method uses to evaluate the SIFs, and the maximum circumferential stress theory uses to calculate the crack growth angles. The crack propagation of two case studies is simulated with the help of the developed program, which uses an adaptive finite element mesh generation approach. The predicted values of the stress intensity factors agreed closely with the available numerical results. During the crack propagation, a particular criterion of the Crack Growth increment utilizes the magnitude of the crack increment. In addition, the Paris law’s expression calculates the fatigue life. Depending on the position of the hole from the crack tip, holes act as crack stoppers and cause cracks to propagate toward them. The program’s results have been verified by comparing them directly to the relevant experimental data and numerical simulations conducted by other researchers.

## Data Availability

The data presented in this study are available upon request from the corresponding author.
